# A Pan-Cancer Analysis of the Oncogenic Role of CD276 in Human Tumors

**DOI:** 10.3390/genes15121527

**Published:** 2024-11-27

**Authors:** Lilong Liu, Zhipeng Yao, Yiting Liu, Yang Li, Yuhong Ding, Junyi Hu, Zhenghao Liu, Pengjie Shi, Ke Chen, Zheng Liu, Wenhao Zhang, Yaxin Hou

**Affiliations:** 1 Department of Urology, Tongji Hospital, Tongji Medical College, Huazhong University of Science and Technology, Wuhan 430030, China; 2 Rehabilitation Medicine Center, The Affiliated Hospital of Hubei Provincial Government, Wuhan 430071, China; crrc100@gmai.com

**Keywords:** CD276, pan-cancer analysis, immunosuppressive, tumor microenvironment, tumor progression

## Abstract

**Objectives**: B7 homolog 3 protein (B7-H3, also known as CD276) is a member of the B7 family that has been found to be associated with the growth and progression of a variety of tumors, but no pan-cancer evaluations of CD276 have been performed so far. In this study, we aimed to perform a pan-cancer analysis of the oncogenic role of CD276 in human tumors; **Methods**: We used a series of databases to perform a pan-cancer analysis of CD276, including the expression level of CD276 in pan-cancer and its relationship to tumor progression, patient survival duration, the immune cell infiltration within the tumor, and the potential signaling pathways and molecular mechanisms associated with CD276; **Results**: We found that CD276 was a potential biomarker for the prognosis of most cancers. The high expression of CD276 was associated with tumor progression, leading to poor survival. Notably, the up-regulation of CD276 expression in tumors increased the tumor infiltration of cancer-associated fibroblasts (CAFs) and myeloid-derived suppressor cells (MDSCs) and decreased the CD8+ T cells; **Conclusions**: Our study demonstrates that CD276 might promote tumor progression via the promotion of an immunosuppressive microenvironment.

## 1. Introduction

Cancer refers to an imbalance between cell proliferation and apoptosis in the body, leading to uncontrolled cell growth, and can be classified according to the cell origin of an organ or tissue [1]. With the rapid development of molecular biology and the popularization of sequencing technology in recent years, increasing the number of cancer subcategories based on the molecular characteristics of cancer cells has been proposed, which broadens people’s understanding of tumorigenesis and tumor progression. This lays a theoretical foundation for developing more accurate diagnosis schemes and more effective and/or less toxic cancer treatment strategies. In the long run, it is necessary to explore the expression and functional changes in each tumor-related gene in pan-cancer so as to provide the best treatment for patients.

B7 homolog 3 protein (B7-H3, also known as CD276) is an identified member of the B7 family. It was first reported as a costimulatory molecule for T-cell activation and Interferon-gamma (IFN-γ) production in 2001 [2]. However, an increasing number of studies have shown that CD276 was frequently overexpressed in tumor cells and tumor vessels of human lung, breast, colon, endometrial, renal, and ovarian cancer [3,4,5,6]. Recent studies have shown that CD276 acted as a T-cell inhibitor to promote the proliferation, invasion, and tumor progression of cancers [7,8,9]. Currently, the potential therapeutic effect of inhibiting CD276 on cancers has attracted people’s attention. Over the past 20 years, many scholars have devoted themselves to identifying the ligands or receptors of CD276 to analyze its physiological function [10,11,12,13,14,15]. Unfortunately, the ligands or receptors of CD276 have not been identified so far.

In this study, we used a series of databases to perform a pan-cancer analysis of CD276, including the expression level of CD276 in pan-cancer and its relationship with tumor progression, patient survival duration, and immune cell infiltration. In addition, we further analyzed the potential proteins interacting with CD276 and genes correlating with its expression, and explored the potential signaling pathways and molecular mechanisms associated with CD276.

## 2. Materials and Methods

### 2.1. Genomic Mapping and Expression Analysis

Genomic localization of the CD276 gene and its expression in 54 tissues from GTEx RNA-seq of 17,382 samples, 948 donors (Release V8) were obtained from the UCSC Genome Browser on Human (GRCh38/hg38, available at http://genome.ucsc.edu/ (accessed on 9 October 2023)) [16]. The RNA tissue specificity of CD276 was analyzed through The Human Protein Atlas (HPA) database (https://www.proteinatlas.org/ (accessed on 9 October 2023)); the consensus normalized expression (NX) ≥1 in at least one tissue type but not being elevated in any tissue type was considered to be low tissue specificity [17]. The online database PanglaoDB (https://panglaodb.se/#google_vignette (accessed on 9 October 2023)) [18] was used to explore the expression of CD276 RNA in different single-cell datasets. The TIMER2.0 web server (http://timer.cistrome.org/ (accessed on 9 October 2023)) [19] and GEPIA2 web server (http://gepia2.cancer-pku.cn/#analysis (accessed on 9 October 2023)) [20] were used to analyze the expression difference in CD276 RNA between tumor and adjacent normal tissues as well as the CD276 expression level in different pathological stages of pan-cancer. Additionally, the expression level of total protein or phosphoprotein (with phosphorylation at the S525 site) of CD276 between tumor and normal tissues in available datasets were explored through the UALCAN web resource (http://ualcan.path.uab.edu/ (accessed on 9 October 2023)) [21].

### 2.2. Prediction of Phosphorylation Sites

The phosphorylation sites of the CD276 protein were explored using the PhosphoNET website, the largest database of known and predicted information on human phosphorylation sites, available at http://www.phosphonet.ca/ (accessed on 9 October 2023).

### 2.3. Survival and Genomic Alteration Analysis

The survival maps and Kaplan–Meier curves of overall survival (OS) and disease-free survival (DFS, also called relapse-free survival, RFS) of CD276 in pan-cancer from the TCGA and the GTEx databases were obtained from the “Survival Analysis” module of the GEPIA2 web server (http://gepia2.cancer-pku.cn/#analysis) (accessed on 10 October 2023) [20]. The web-based tool Kaplan–Meier plotter (http://kmplot.com/analysis/ (accessed on 10 October 2023) ) [22] was used to generate the OS for breast cancer, ovarian cancer, lung cancer, gastric cancer, and liver cancer from the GEO, EGA, and TCGA databases. Herein, cBioPortal (http://www.cbioportal.org/ (accessed on 10 October 2023)) [23,24] was used to explore the effect of CD276 mutation on the OS, DFS, disease-specific survival (DSS), and progression-free survival (PFS) of patients with skin cutaneous melanoma (SKCM), uterine corpus endometrial carcinoma (UCEC), or colorectal adenocarcinoma (COAD). The genomic alteration analysis of CD276 was conducted through the cBioPortal web resource (https://www.cbioportal.org/ (accessed on 10 October 2023)) [23,24]. In brief, we obtained the genomic alteration frequency and alteration type of CD276 in pan-cancer from the “Cancer Types Summary” module. Via the “Mutations” module, we analyzed the spatial distribution of each mutation site, the case number of the CD276 mutation, and the proportion of each cancer species in patients with CD276 mutation. Moreover, the 3D structure of CD276 was generated by the “Mutations” module and the site with the most frequent mutation was marked.

### 2.4. Exploration of Immune Infiltration

The “Immune” module of the TIMER2.0 web server (http://timer.cistrome.org/ (accessed on 11 October 2023)) [19] was used to visualize the correlation (Spearman correlation coefficient) of CD276 expression with immune infiltration level in pan-cancer. Cancer-associated fibroblasts (CAFs), myeloid-derived suppressor cells (MDSCs), and CD8+ T cells have a strong correlation with the expression of CD276 and are relatively better at illustrating the relationship between CD276 and immune suppression, we chose them for presentation. In addition, a linear regression of CD276 expression level and tumor purity or CAFs_TIDE (CAFs_Tumor Immune Dysfunction and Exclusion) were downloaded. The CAFs_TIDE results were adjusted based on tumor purity.

### 2.5. Functional Analysis of CD276

Thirteen experimentally validated CD276-binding proteins were obtained from the STRING Database [25]. The confidence threshold for the interaction network was set at 0.4. The top 100 CD276-correlated gene list was generated through the “Similar Genes Detection” module of GEPIA2 (http://gepia2.cancer-pku.cn/#analysis (accessed on 11 October 2023)) [20]. The protein–protein interaction (PPI) networks were generated from the STRING database; the query list included either 13 (CD276-binding proteins) or 113 (CD276-binding proteins plus CD276-correlated genes) items [25]. Apart from that, the CD276-correlated genes and CD276-binding proteins were used to perform Gene ontology (GO) enrichment analysis and *Kyoto Encyclopedia of Genes and Genomes* (KEGG) pathway analysis by the database for annotation, visualization, and integrated discovery (DAVID) [26] and R statistical software (version 4.1.1).

### 2.6. Western Blot

Cells with good growth status, including SV-1, EJ, T24, HK2, 786O, and A498, were collected. Proteins were extracted, and the protein concentration was measured using the BCA method. The protein concentration was standardized to 3 µg/µL for Western blot analysis. The CD276 antibody used was CD276 Rabbit mAb (Catalog No. R381871, Zhengneng Biotech, Chengdu, China, diluted 1:1000), and the β-actin antibody used was β-actin Rabbit mAb (Catalog No. AC026, Abclonal, Wuhan, China, diluted 1:3000).

## 3. Results

### 3.1. Chromosome Localization and Expression Distribution of CD276

As shown in Figure S1A, the human CD276 gene is located in chromosome 15 (q24.1). The CD276 gene was expressed in 54 tissues from GTEx (Release V8) [16], and the highest expression was found in cultured fibroblasts (Figure S1B). In the HPA database [17], we found that CD276 was expressed in all available tissues (NX ≥ 1), indicating that CD276 has low tissue specificity (Figure S1C). Moreover, we found that CD276 was mainly expressed in endothelial cells, basal cells, fibroblasts, and immune cells at the single-cell level (Figure S2A and Supplementary Table S1).

**Figure 1:**
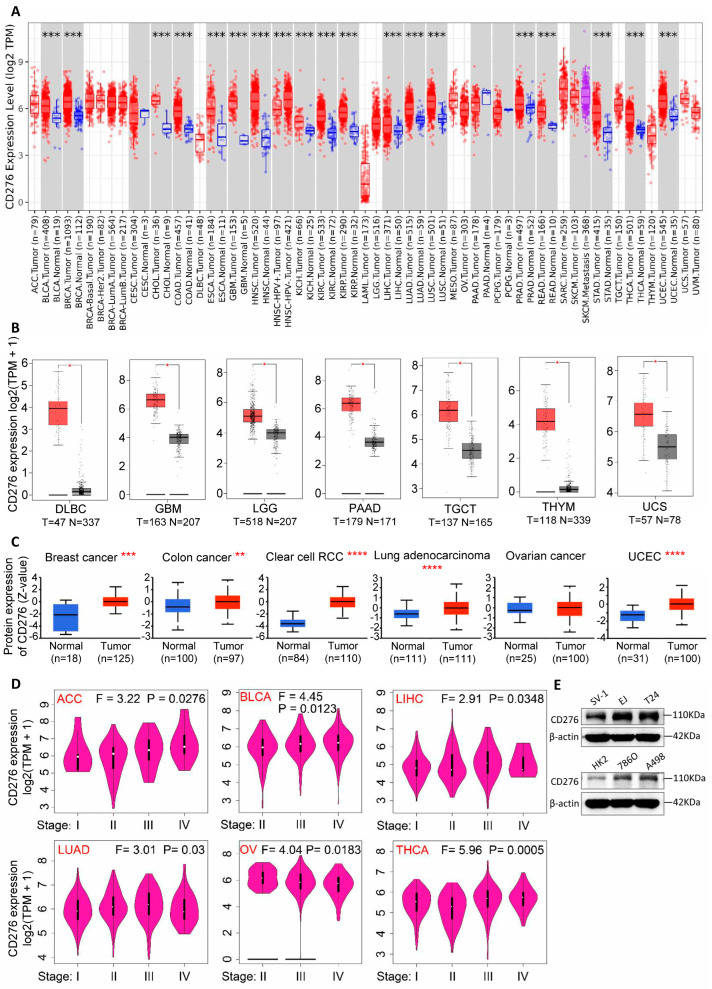
Expression level of CD276 in pan-cancer and pathological stages. The expression level of CD276 mRNA (**A**,**B**) and protein (**C**) in different tumor and adjacent normal tissues. (**D**) The CD276 expression level in different pathological stages of pan-cancer from the TCGA database. (**E**) The CD276 protein expression level of SV-1, EJ, T24, HK2, 7860, and A498 as detected by Western blot. * *p* < 0.05, ** *p* < 0.01, *** *p* < 0.001, **** *p* < 0.0001.

To explore the relationship between the expression level of CD276 and cancers, both the TIMER2.0 [19] and GEPIA2 web server [20] were used. As shown in Figure 1A,B and Figure S2B, the expression level of CD276 RNA in the tumor tissues of BLCA, BRCA, CHOL, COAD, ESCA, GBM, HNSC, KICH, KIRC, KIRP, LIHC, LUAD, LUSC, PRAD, READ, STAD, THCA, UCEC, DLBC, LGG, PAAD, TGCT, THYM, and UCS (*p* < 0.05), but excluding ACC, CESC, LAML, OV, and PCPG (*p* > 0.05), were significantly higher than those in the adjacent normal tissues (an explanation of the abbreviations is shown in Supplementary Table S2). Similarly, the analysis of available datasets from the UALCAN web resource [21] showed that the expression of CD276 total protein in BRCA, COAD, KIRC, LUAD, and UCEC was dramatically higher than that in adjacent normal tissues (Figure 1C). We performed validation in both bladder cancer and renal cancer cell lines and similarly found that CD276 expression in tumor cells was significantly higher than in normal urothelial cells and renal tubular epithelial cells (Figure 1E). Meanwhile, breast cancer, ovarian cancer, and colon cancer (available datasets) also exhibited higher levels of the CD276 protein phosphorylated at residue S525 (Figure S2C). In addition, we found that the expression level of CD276 was related to tumor stage in ACC, BLCA, LIHC, LUAD, THCA, and OV (Figure 1D and Figure S2D). Therefore, CD276 is highly expressed in tumor tissues and is associated with tumor progression.

### 3.2. Correlation Between Expression Level and Survival Prognosis of Patients with or Without Genomic Alteration of CD276

**Figure 2:**
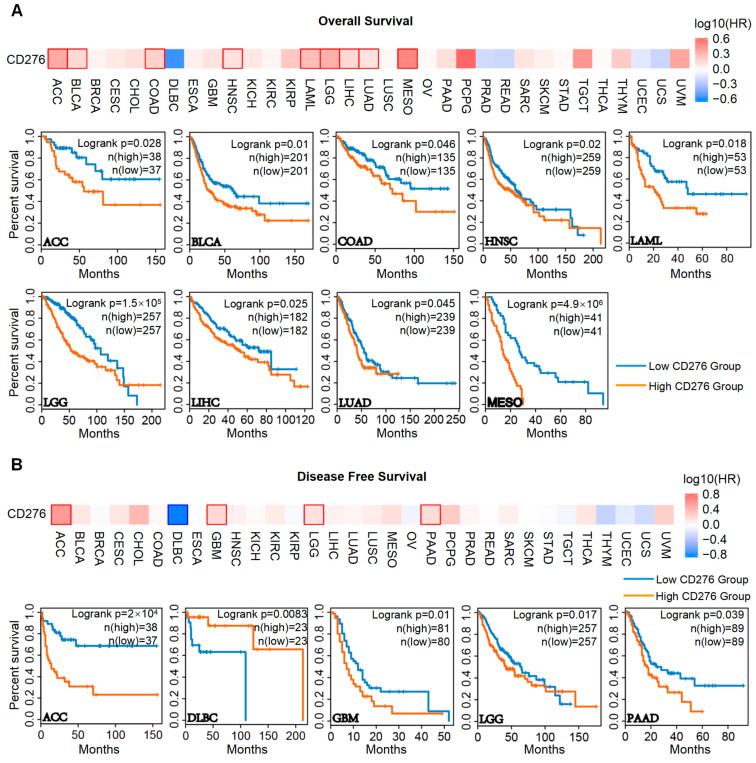
Correlation between CD276 expression level and the survival prognosis of pan-cancer. Using the GEPIA2 web server, we found that increased expression of CD276 forecasted a shorter overall survival (**A**) in the patients with ACC, BRCA, BLCA, COAD, HNSC, LAML, LGG, LIHC, LUAD, and MESO, and a shorter disease-free survival (**B**) in patients with ACC, GBM, LGG, and PAAD. We presented the survival map and Kaplan–Meier curves with positive results.

**Figure 3:**
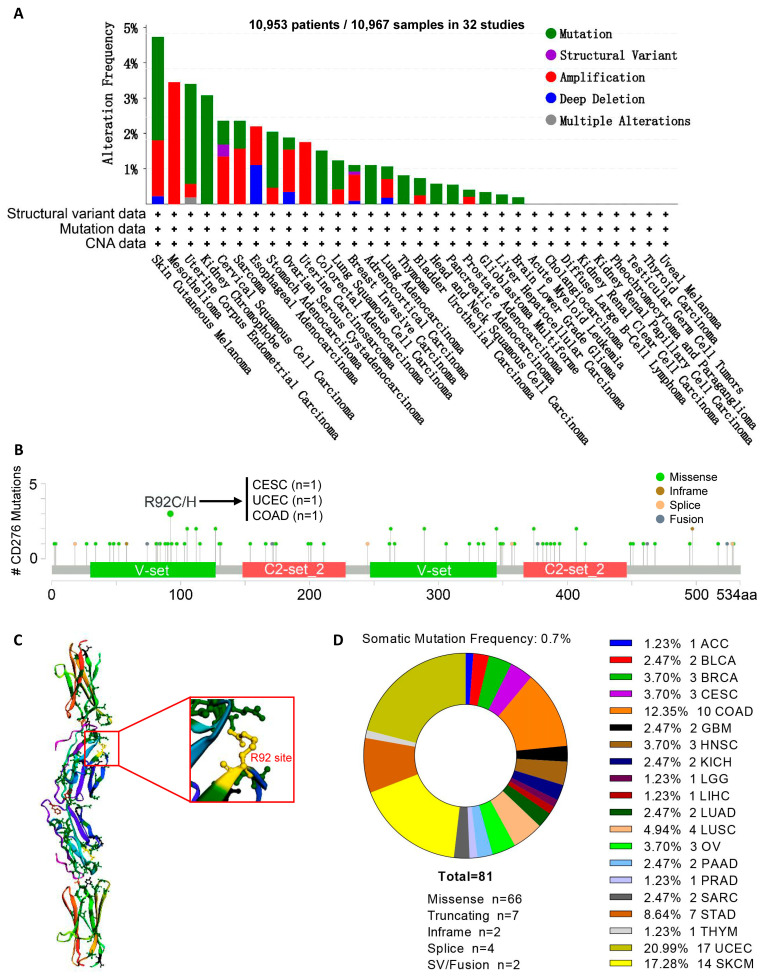
The genomic alteration of CD276 in pan-cancer. We used the cBioPortal web resource to explore the genomic alteration of CD276 in pan-cancer. (**A**) The genomic alteration frequency and alteration type of CD276 in pan-cancer. (**B**) The spatial distribution of each mutation site and case number of the CD276 mutation. (**C**) The 3D structure of CD276 with the most frequent mutation site labeled. (**D**) The proportion of each cancer species in patients with CD276 mutation.

To explore the relationship between the expression of CD276 and the prognosis of patients, we employed the GEPIA2 web server [20] and the Kaplan–Meier plotter [22] and found that increased expression of CD276 forecasted a shorter OS in patients with ACC, BRCA, BLCA, COAD, HNSC, LAML, LGG, LIHC, LUAD, MESO (Mesothelioma), gastric cancer, and ovarian cancer, and a shorter DFS in patients with ACC, GBM, LGG, and PAAD (Figure 2A,B and Figure S3A). However, DLBC (Diffuse Large B-Cell Lymphoma) showed an opposing trend compared to ACC, GBM, LGG, and PAAD, which warrants further investigation. To explore the genomic alteration of CD276 in pan-cancer, the cBioPortal web resource [23,24] was used. As shown in Figure 3A, patients with SKCM had the highest genomic alteration frequency, followed by MESO, UCEC, and KICH. The detailed spatial distribution and case number of each mutation site of CD276 are presented in Figure 3B. We found that R92C/H alteration within the V-set domain was the most common. The 3D structure of CD276 with R92 site labeling is displayed in Figure 3C. Among all patients with mutations in CD276, patients with UCEC, COAD, and SKCM accounted for the highest proportion (Figure 3D). Subsequently, we explored the impact of CD276 mutations on the OS, DFS, DSS, and PFS of patients with SKCM, UCEC, or COAD. The results showed that the survival time of patients with CD276 mutations tended to be prolonged, but there was no significant difference (Figure S3B). Collectively, the increased expression of CD276 in tumor tissue promotes tumor progression, and targeting CD276 might be able to prolong the survival time of patients.

### 3.3. Effect of High Expression of CD276 on Immune Cell Infiltration

**Figure 4:**
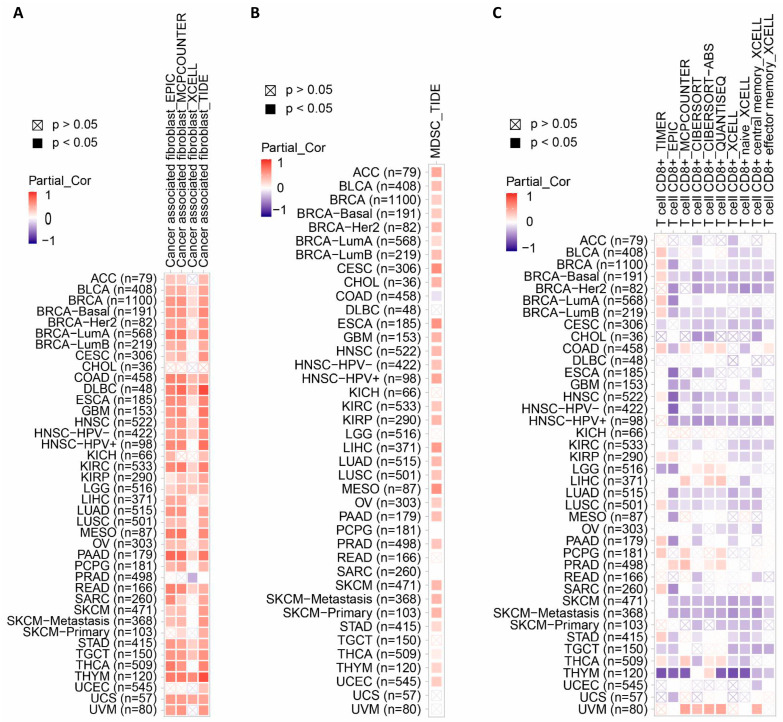
The effect of the high expression of CD276 on immune cell infiltration. A remarkable positive correlation between CD276 expression and the immune infiltration of cancer-associated fibroblasts (**A**) and myeloid-derived suppressor cells (**B**) in pan-cancer. (**C**) A significant negative correlation between the CD276 expression and immune infiltration of CD8+ T cells in pan-cancer.

To explore the regulation of CD276 on the tumor microenvironment (TME), we further analyzed the correlation between the expression level of CD276 and immune cell infiltration using the TIMER2.0 web server with the EPIC, MCPCOUNTER, XCELL, TIDE, TIMER, CIBERSORT, CIBERSORT-ABS, and QUANTISEQ algorithms [19]. It was observed that there was a remarkable positive correlation between CD276 expression and the immune infiltration of CAFs and MDSCs in almost all tumors (Figure 4A,B). The linear regression of CD276 expression level and tumor purity and the immune infiltration of CAFs_TIDE are shown in Figure S4 as well as Figure S5. Moreover, the results show a significant negative correlation between CD276 expression and the immune infiltration of CD8+ T cells in BLCA, BRCA, CESC, HNSC, HNSC-HPV+, KIRC, LUAD, LUSC, OV, SKCM, SKCM-Metastasis, STAD, TGCT, and THYM, based on all or most algorithms (Figure 4C). Previous studies [27] have identified a major role of CAFs in the establishment of an immunosuppressive TME by recruiting immunosuppressive populations, such as MDSCs and neutrophils, and secreting immunosuppressive ligands, such as TGF-β and CXCL12. Similarly, MDSCs also hindered the anti-tumor roles of many immune cells, such as Natural Killer (NK) cells, B cells, and T cells [28]. These results indicate that the expression of CD276 is involved in the formation of immunosuppressive TME and hinders the anti-tumor immune response.

### 3.4. Analysis of CD276-Binding Proteins and Functional Pathways

**Figure 5:**
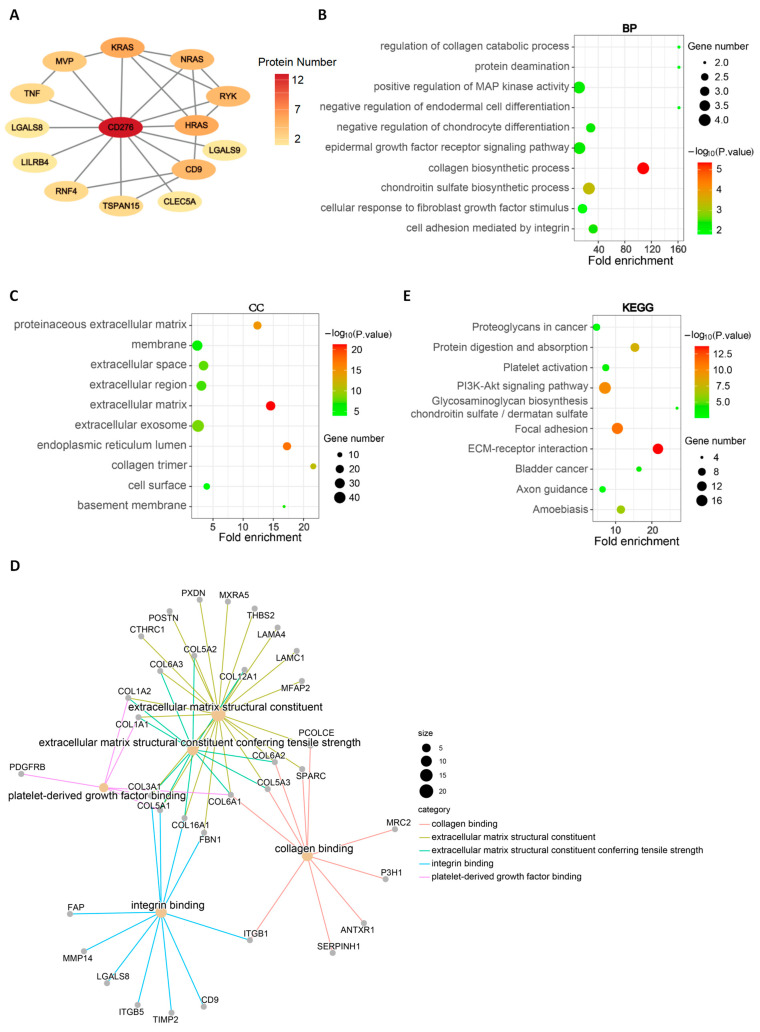
Analysis of CD276-binding proteins and functional pathways. (**A**) The protein–protein interaction network of CD276-binding proteins. The biological process (**B**), cellular component (**C**), and molecular functions (**D**) of CD276-correlated genes and binding proteins in enrichment analysis of the GO pathway. (**E**) The enrichment pathways of CD276-correlated genes and binding proteins in KEGG pathway analysis.

To further investigate the function of CD276, we obtained 13 experimentally validated CD276-binding proteins from the STRING database [25], i.e., LGALS8, LGALS9, CD9, HRAS, NRAS, KRAS, RYK, MVP, TNF, LILRB4, RNF4, TSPAN15, and CLEC5A. The PPI network of CD276-binding proteins is shown in Figure 5A. Next, a list of 100 CD276-correlated genes were downloaded through the GEPIA2 web server [20].

This resulted in 113 nodes interacting with CD276; then, we combined the CD276-correlated genes with the CD276-binding proteins for subsequent analysis. As shown in Figure S6, collagen-related genes were the most prominent hub genes among the interaction network of CD276-correlated genes and binding proteins. GO enrichment analysis further showed that most of these genes are related to extracellular matrix structural constituents, collagen biosynthetic processes, and cell adhesion, including the proteinaceous extracellular matrix, the extracellular matrix, the extracellular region, the extracellular exosome, the collagen trimer, collagen binding, cell adhesion mediated by integrin, integrin binding, the cellular response to fibroblast growth factor stimulus, and positive regulation of MAP kinase activity (Figure 5B–D). Additionally, the KEGG enrichment analysis indicated that “Proteoglycans in cancer”, “Focal adhesion”, “ECM-receptor interaction”, and the “PI3K-Akt signaling pathway” might be involved in CD276-mediated TME reconstruction (Figure 5E). Overall, CD276 might mediate the reconstruction of TME by influencing various signaling pathways, and promoting tumorigenesis and tumor progression.

## 4. Discussion

Although great advances have been made in the diagnosis and treatment of cancer, cancer still threatens the survival of mankind. Therefore, it is urgent to further analyze the mechanism of tumorigenesis and tumor progression, which should result in new strategies for the treatment of cancer. Since CD276 was first reported on in 2001 [2], many achievements have been accomplished over the past 20 years. Accumulating evidence suggests that CD276 is an immunosuppressive checkpoint [7,8] rather than a T-cell costimulator, as previously thought [2]. Recently, numerous studies have shown that the expression of CD276 in prostate cancer, breast cancer, and other tumors is significantly up-regulated, and it is directly associated with tumor avoidance of the host immune response and the poor prognosis of these patients [3,4,5,6,29,30,31,32]. To date, anti-CD276 antibodies have been shown to significantly promote the infiltration of CD8+ T cells and inhibit the growth of tumors [8,29,33]. However, the ligands or receptors of CD276 and the physiological function of CD276 have yet to be discovered [10,11,12,13,14].

In this study, we demonstrated that CD276 was expressed in 54 tissues with a low tissue specificity but highly expressed in basal cells, fibroblasts, and immune cells. Using the TIMER2.0 [19], GEPIA2 [20], and UALCAN web resources [21], we found that the expression level of CD276 in 24 tumor tissues was significantly higher than that in adjacent normal tissues. This is consistent with previous studies reporting the increased expression of CD276 in various tumors [7,8,30]. Although we found that CD276 expression in LAML is lower than in other tumors, there is no normal control data for LAML in TIMER2.0. Furthermore, related studies have reported that an increase in CD276 expression is correlated with tumor progression [34,35,36], and we confirmed this in ACC, BLCA, LIHC, LUAD, THCA, and OV. Survival analysis showed that up-regulation of CD276 expression is associated with a poor prognosis based on OS and DFS endpoints. Taken together, CD276 is highly expressed in the vast majority of tumors and is associated with tumor progression, which indicates its potential in tumor therapy.

To further explore the regulation of CD276 on TME, a correlation analysis between CD276 expression and immune infiltration was performed. We found that the up-regulation of CD276 expression increased the infiltration of CAFs and MDSCs in tumors, and decreased CD8+ T cells. Previous studies [27,28,37,38,39] have proven that CAFs and MDSCs are among the main factors in the formation of an immune-inhibiting TME, indicating that CD276 is involved in the inhibition of anti-tumor immune response. Thus, CD276 is considered to be an attractive and promising tumor immunotherapy target, and plenty of related CD276-targeting drugs are currently undergoing or have completed clinical trials [7]. It has been demonstrated that blocking CD276 with monoclonal antibodies increases the tumor infiltration of CD8+ T cells and NK cells, reduces tumor growth, and prolongs survival in different mouse tumor models [40,41,42].

Finally, PPI network and enrichment analyses were performed to investigate the function of CD276. We obtained 13 experimentally validated CD276-binding proteins from the STRING database [25] and the results of enrichment analysis further showed that most CD276-related genes are involved in the pathways of the extracellular matrix structural constituent, ECM–receptor interaction, collagen biosynthetic process, cell adhesion mediated by integrin, and cellular response to fibroblast growth factor stimulus. This was consistent with the high expression of CD276 in stromal cells and fibroblasts. Therefore, we speculated that CD276 promoted the formation of an immunosuppressive microenvironment and ultimately promoted the progression of tumors by interacting with potential ligands or receptors in the TME.

## 5. Conclusions

In conclusion, CD276 has been discovered to be a potential prognostic biomarker for pan-cancer. An increase in CD276 expression is correlated with tumor progression and poor survival. In addition, the up-regulation of CD276 expression increases the infiltration of CAFs and MDSCs in tumors, but decreases those of CD8+ T cells. CD276 is involved in tumor progression and affects the TME, making it more immunosuppressive.

## Data Availability

The original contributions presented in the study are included in the article/Supplementary Material, further inquiries can be directed to the corresponding author.

## Supplementary Materials

The following supporting information can be downloaded at: https://www.mdpi.com/article/10.3390/genes15121527/s1, Figure S1: Chromosome localization and expression distribution of CD276; Figure S2: Expression level of CD276 in pan-cancer and pathological stages; Figure S3: Correlation between expression level and survival prognosis of patients with or without genomic alteration of CD276; Figures S4 and S5: A linear regression of CD276 expression level and tumor purity or the immune infiltration of cancer associated fibroblasts; Figure S6: The protein–protein interaction network of the CD276-correlated genes and binding proteins showed that collagen-related genes were the most prominent hub gene; Table S1: The expression of CD276 mRNA in different single cells. Table S2: Cancer Types and Abbreviations Table.
