# Supplementary material for: A Pan-Cancer Analysis of the Oncogenic Role of CD276 in Human Tumors
Source: Genes (Basel). 2024 Nov 27;15(12):1527. doi: 10.3390/genes15121527 (PMC11675885; doi:10.3390/genes15121527)
Supplement: Supplementary file 1 [file genes-15-01527-s001.zip › genes-3300171-supplementary.pdf]

Figure S1

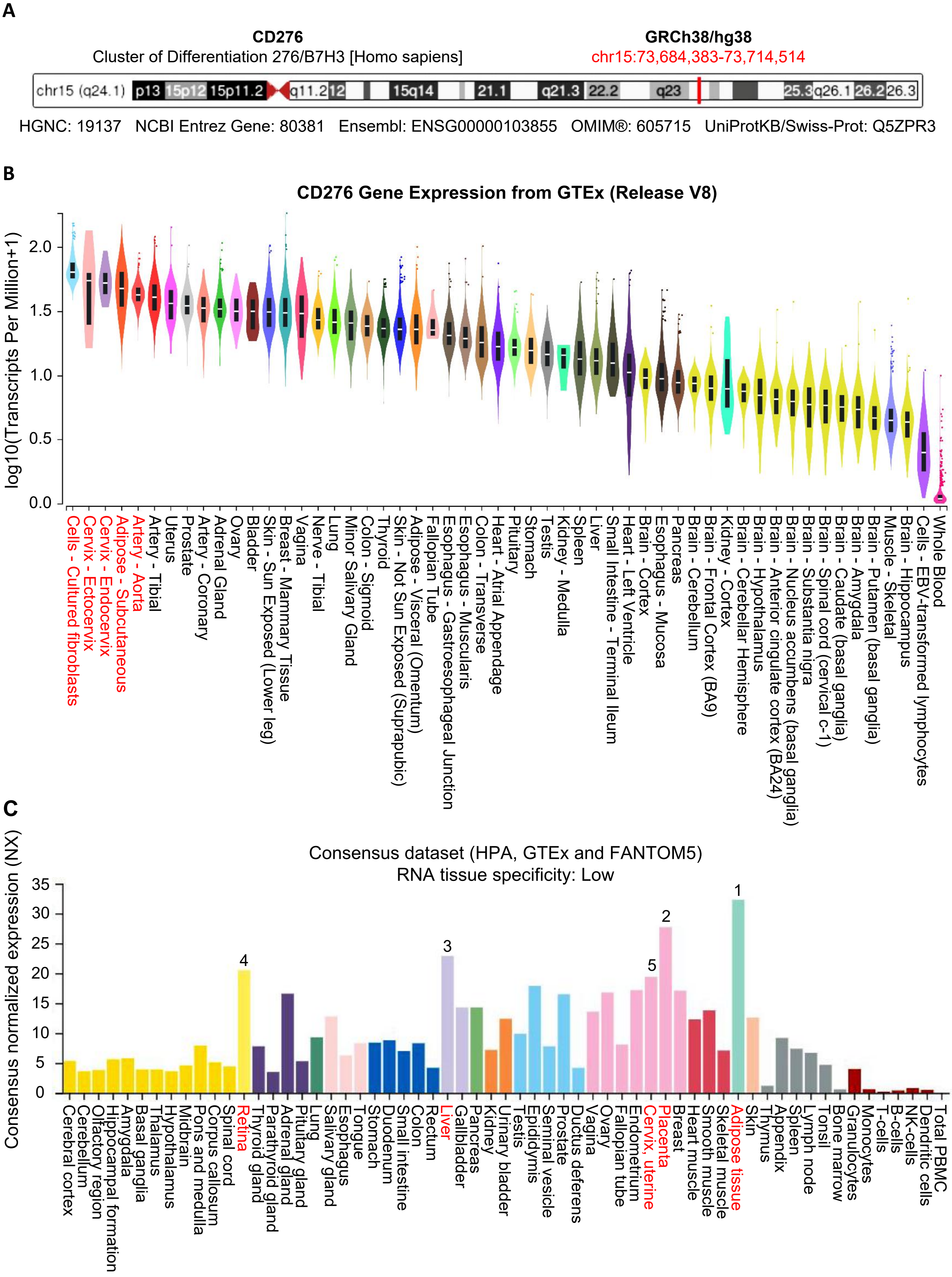
A
CD276
Cluster of Differentiation 276/B7H3 [Homo sapiens]
GRCh38/hg38
chr15:73,684,383-73,714,514
chr15 (q24.1)
p13
15p12
15p11.2
q11.2
12
15q14
21.1
q21.3
22.2
q23
25.3
q26.1
26.2
26.3
HGNC: 19137 NCBI Entrez Gene: 80381 Ensembl: ENSG00000103855 OMIM®: 605715 UniProtKB/Swiss-Prot: Q5ZPR3
B
CD276 Gene Expression from GTEx (Release V8)
log10(Transcripts Per Million+1)
0.0
0.5
1.0
1.5
2.0
Cells - Cultured fibroblasts
Cervix - Ectocervix
Cervix - Endocervix
Adipose - Subcutaneous
Artery - Aorta
Artery - Tibial
Uterus
Prostate
Artery - Coronary
Adrenal Gland
Ovary
Bladder
Skin - Sun Exposed (Lower leg)
Breast - Mammary Tissue
Vagina
Nerve - Tibial
Lung
Minor Salivary Gland
Colon - Sigmoid
Thyroid
Skin - Not Sun Exposed (Suprapubic)
Adipose - Visceral (Omentum)
Fallopian Tube
Esophagus - Gastroesophageal Junction
Esophagus - Muscularis
Colon - Transverse
Heart - Atrial Appendage
Pituitary
Stomach
Testis
Kidney - Medulla
Spleen
Liver
Small Intestine - Terminal Ileum
Heart - Left Ventricle
Brain - Cortex
Esophagus - Mucosa
Pancreas
Brain - Cerebellum
Brain - Frontal Cortex (BA9)
Kidney - Cortex
Brain - Cerebellar Hemisphere
Brain - Hypothalamus
Brain - Anterior cingulate cortex (BA24)
Brain - Nucleus accumbens (basal ganglia)
Brain - Substantia nigra
Brain - Spinal cord (cervical c-1)
Brain - Caudate (basal ganglia)
Brain - Amygdala
Brain - Putamen (basal ganglia)
Muscle - Skeletal
Brain - Hippocampus
Cells - EBV-transformed lymphocytes
Whole Blood
C
Consensus dataset (HPA, GTEx and FANTOM5)
RNA tissue specificity: Low
Consensus normalized expression (NX)
0
5
10
15
20
25
30
35
1
2
3
4
5
Cerebral cortex
Cerebellum
Olfactory region
Hippocampal formation
Amygdala
Basal ganglia
Thalamus
Hypothalamus
Midbrain
Pons and medulla
Corpus callosum
Spinal cord
Retina
Thyroid gland
Parathyroid gland
Adrenal gland
Pituitary gland
Lung
Salivary gland
Esophagus
Tongue
Stomach
Duodenum
Small intestine
Colon
Rectum
Liver
Gallbladder
Pancreas
Kidney
Urinary bladder
Testis
Epididymis
Seminal vesicle
Prostate
Ductus deferens
Vagina
Ovary
Fallopian tube
Endometrium
Cervix, uterine
Placenta
Breast
Heart muscle
Smooth muscle
Skeletal muscle
Adipose tissue
Skin
Thymus
Appendix
Spleen
Lymph node
Tonsil
Bone marrow
Granulocytes
Monocytes
T-cells
B-cells
NK-cells
Dendritic cells
Total PBMC

Figure S2

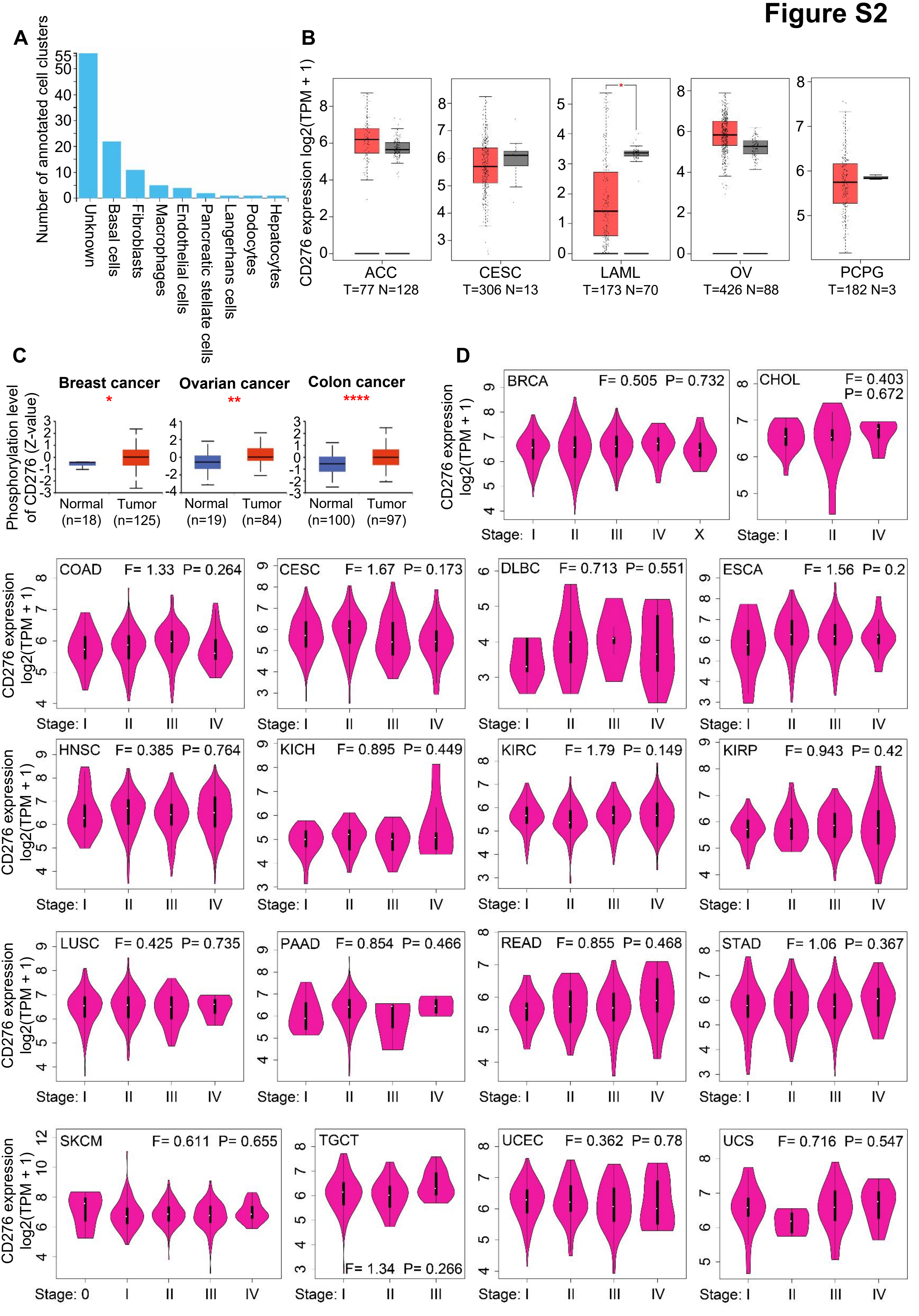

A
Number of annotated cell clusters
Unknown
Basal cells
Fibroblasts
Macrophages
Endothelial cells
Pancreatic stellate cells
Langerhans cells
Podocytes
Hepatocytes
B
CD276 expression log2(TPM + 1)
ACC
T=77 N=128
CESC
T=306 N=13
LAML
T=173 N=70
OV
T=426 N=88
PCPG
T=182 N=3
C
Phosphorylation level of CD276 (Z-value)
Breast cancer
Ovarian cancer
Colon cancer
Normal (n=18)
Tumor (n=125)
Normal (n=19)
Tumor (n=84)
Normal (n=100)
Tumor (n=97)
D
CD276 expression log2(TPM + 1)
BRCA F= 0.505 P= 0.732
CHOL F= 0.403 P= 0.672
COAD F= 1.33 P= 0.264
CESC F= 1.67 P= 0.173
DLBC F= 0.713 P= 0.551
ESCA F= 1.56 P= 0.2
HNSC F= 0.385 P= 0.764
KICH F= 0.895 P= 0.449
KIRC F= 1.79 P= 0.149
KIRP F= 0.943 P= 0.42
LUSC F= 0.425 P= 0.735
PAAD F= 0.854 P= 0.466
READ F= 0.855 P= 0.468
STAD F= 1.06 P= 0.367
SKCM F= 0.611 P= 0.655
TGCT F= 1.34 P= 0.266
UCEC F= 0.362 P= 0.78
UCS F= 0.716 P= 0.547
Stage: I II III IV X
Stage: 0 I II III IV

Figure S3

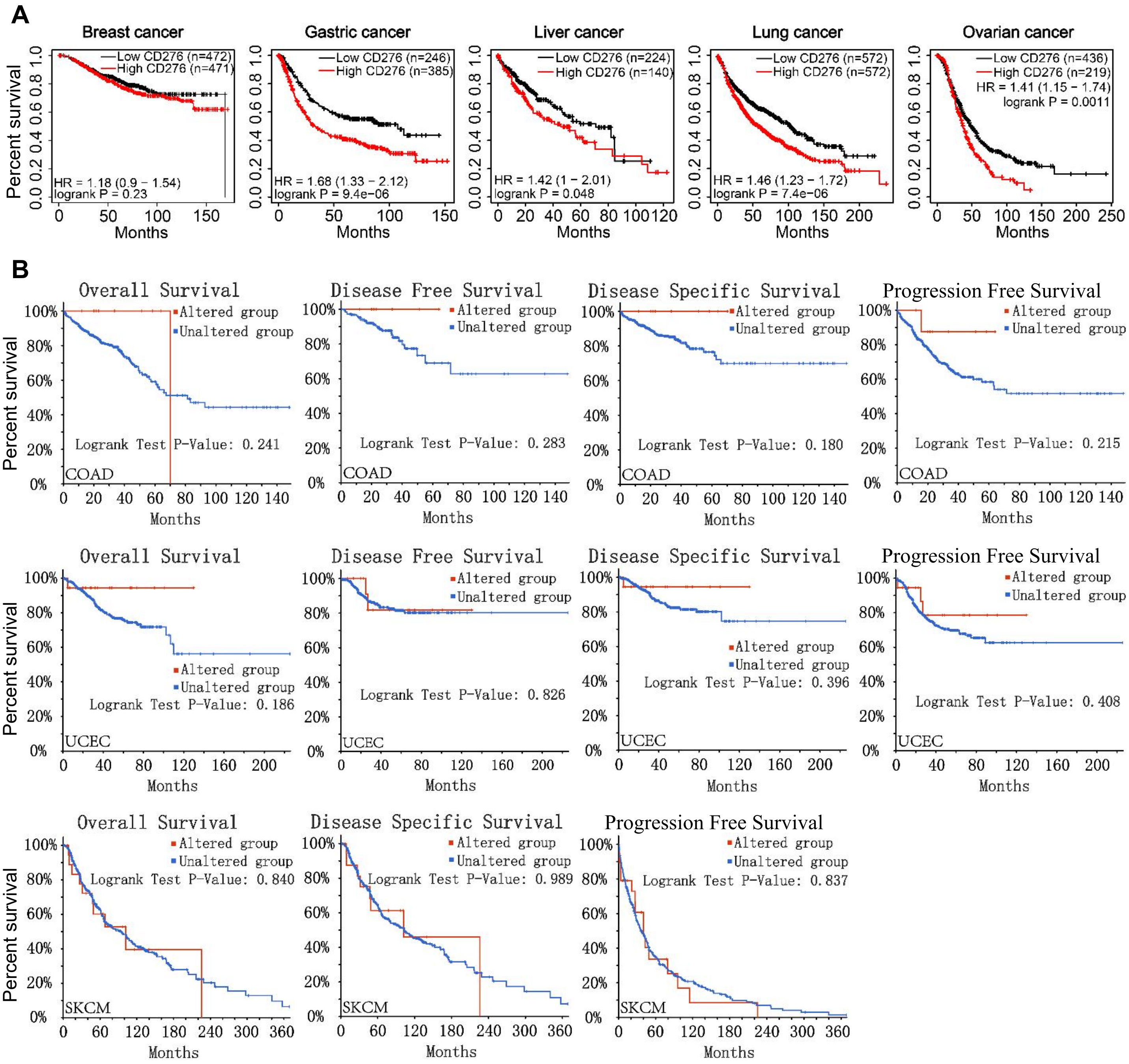
A
Breast cancer
Low CD276 (n=472)
High CD276 (n=471)
HR = 1.18 (0.9 − 1.54)
logrank P = 0.23
Gastric cancer
Low CD276 (n=246)
High CD276 (n=385)
HR = 1.68 (1.33 − 2.12)
logrank P = 9.4e−06
Liver cancer
Low CD276 (n=224)
High CD276 (n=140)
HR = 1.42 (1 − 2.01)
logrank P = 0.048
Lung cancer
Low CD276 (n=572)
High CD276 (n=572)
HR = 1.46 (1.23 − 1.72)
logrank P = 7.4e−06
Ovarian cancer
Low CD276 (n=436)
High CD276 (n=219)
HR = 1.41 (1.15 − 1.74)
logrank P = 0.0011
Percent survival
Months
B
Overall Survival
Disease Free Survival
Disease Specific Survival
Progression Free Survival
Altered group
Unaltered group
Logrank Test P-Value: 0.241
Logrank Test P-Value: 0.283
Logrank Test P-Value: 0.180
Logrank Test P-Value: 0.215
COAD
Logrank Test P-Value: 0.186
Logrank Test P-Value: 0.826
Logrank Test P-Value: 0.396
Logrank Test P-Value: 0.408
UCEC
Logrank Test P-Value: 0.840
Logrank Test P-Value: 0.989
Logrank Test P-Value: 0.837
SKCM
Percent survival
Months

Figure S4

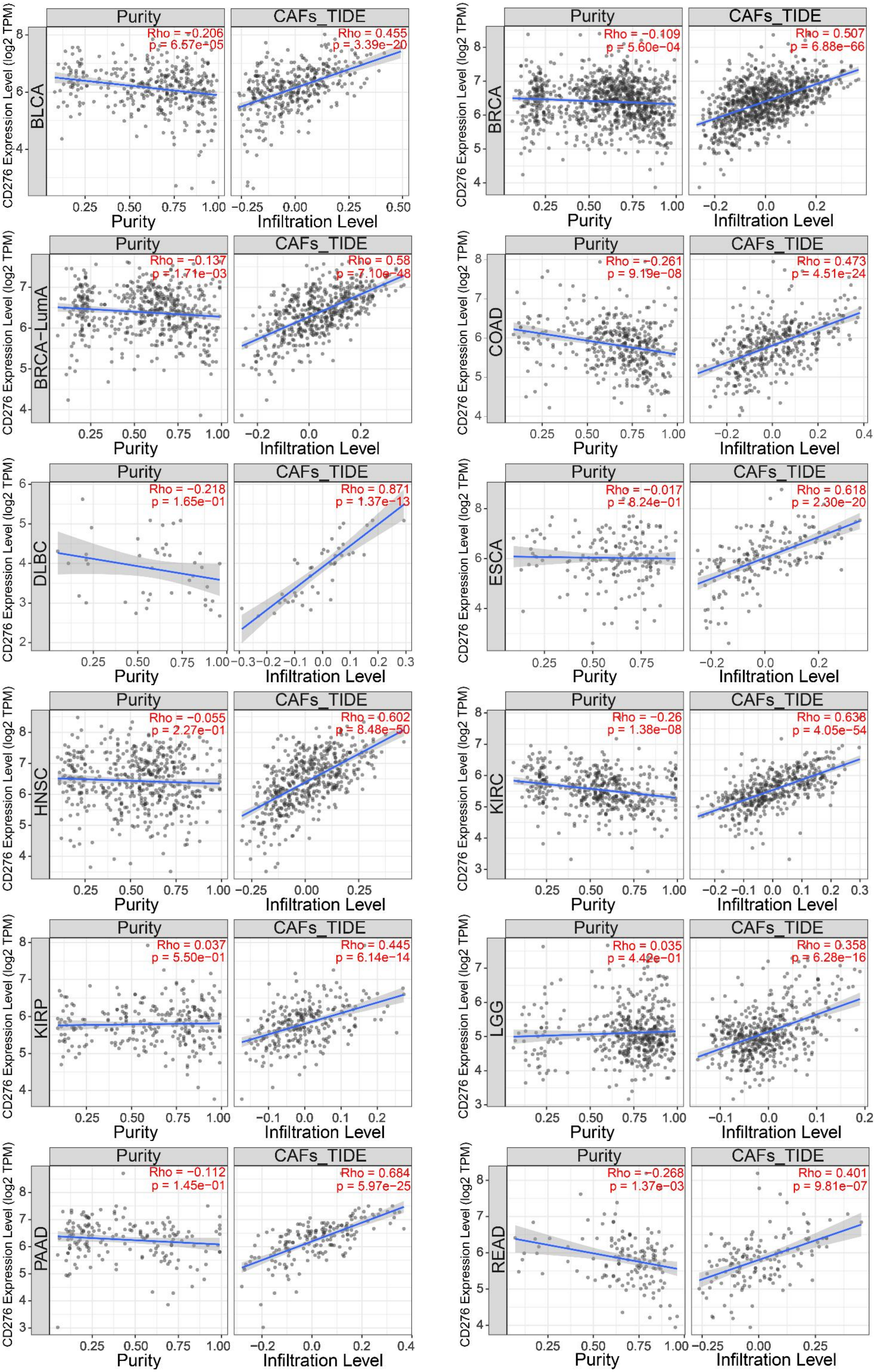
Purity
CAFs_TIDE
CD276 Expression Level (log2 TPM)
Infiltration Level
BLCA
Rho = −0.206
p = 6.57e−05
Rho = 0.455
p = 3.39e−20
BRCA
Rho = −0.109
p = 5.60e−04
Rho = 0.507
p = 6.88e−66
BRCA−LumA
Rho = −0.137
p = 1.71e−03
Rho = 0.58
p = 7.10e−48
COAD
Rho = −0.261
p = 9.19e−08
Rho = 0.473
p = 4.51e−24
DLBC
Rho = −0.218
p = 1.65e−01
Rho = 0.871
p = 1.37e−13
ESCA
Rho = −0.017
p = 8.24e−01
Rho = 0.618
p = 2.30e−20
HNSC
Rho = −0.055
p = 2.27e−01
Rho = 0.602
p = 8.48e−50
KIRC
Rho = −0.26
p = 1.38e−08
Rho = 0.638
p = 4.05e−54
KIRP
Rho = 0.037
p = 5.50e−01
Rho = 0.445
p = 6.14e−14
LGG
Rho = 0.035
p = 4.42e−01
Rho = 0.358
p = 6.28e−16
PAAD
Rho = −0.112
p = 1.45e−01
Rho = 0.684
p = 5.97e−25
READ
Rho = −0.268
p = 1.37e−03
Rho = 0.401
p = 9.81e−07

Figure S5

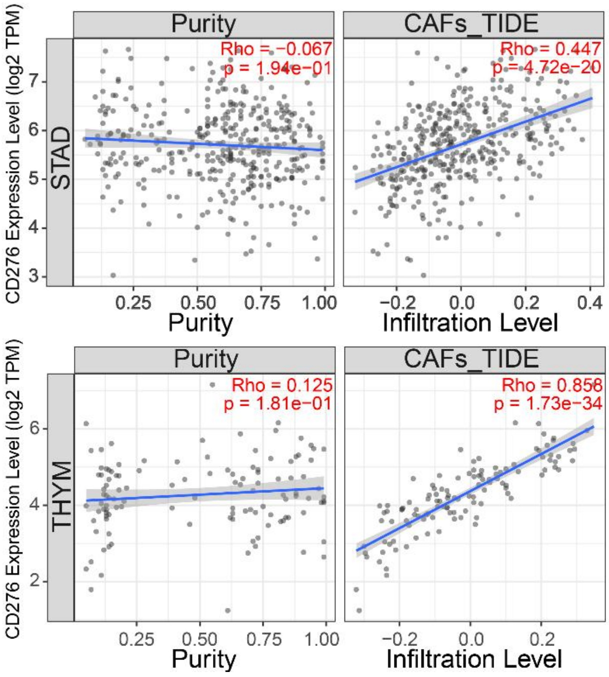
STAD
TGCT
THYM
UCS
Purity
CAFs_TIDE
CD276 Expression Level (log2 TPM)
Infiltration Level
Rho = −0.067
p = 1.94e−01
Rho = 0.447
p = 4.72e−20
Rho = 0.419
p = 1.12e−07
Rho = 0.543
p = 1.17e−12
Rho = 0.125
p = 1.81e−01
Rho = 0.858
p = 1.73e−34
Rho = 0.074
p = 5.96e−01
Rho = 0.462
p = 4.92e−04

Figure S6

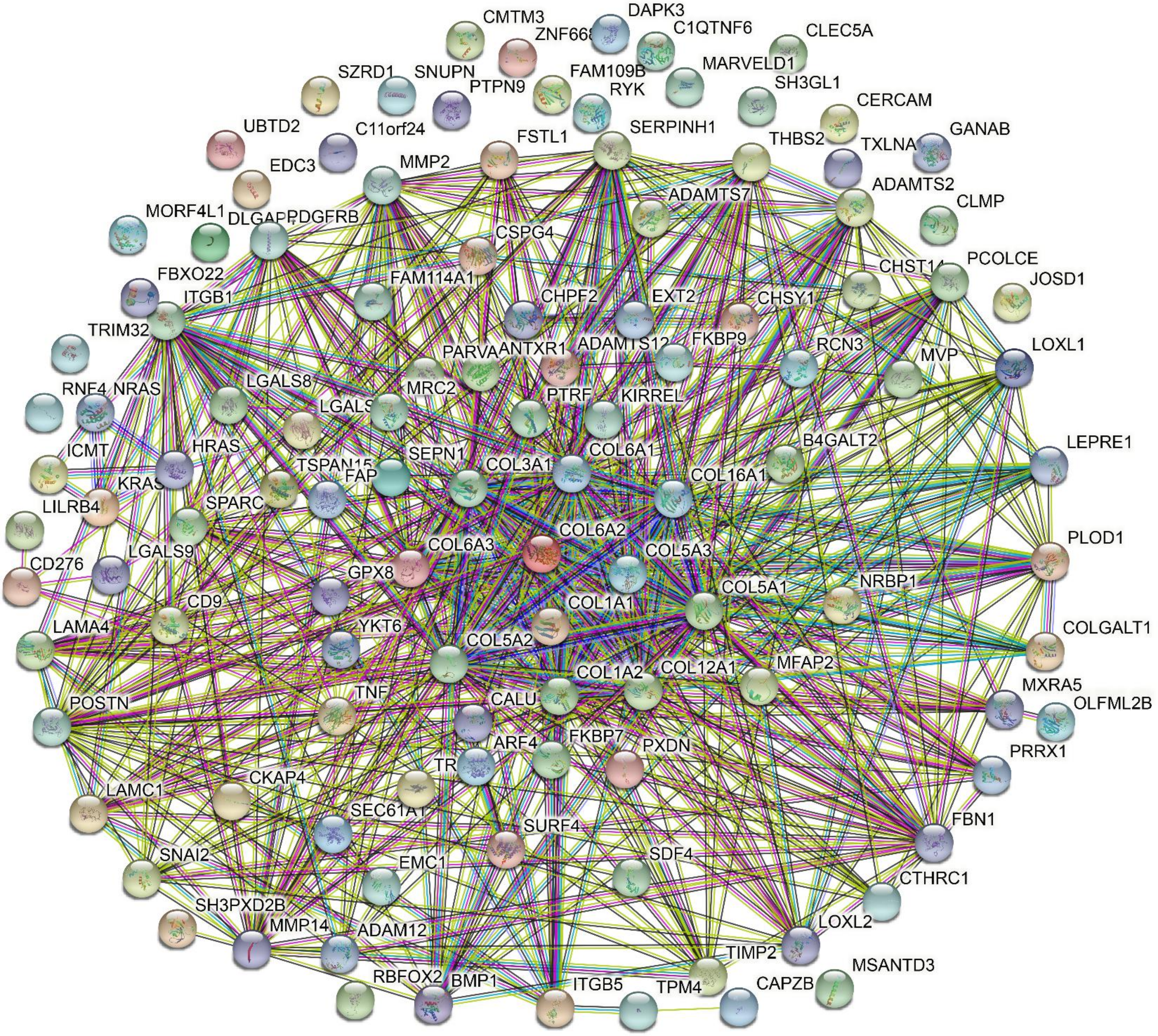
CMTM3
DAPK3
ZNF66
C1QTNF6
CLEC5A
SZRD1
SNUPN
PTPN9
FAM109B
RYK
MARVELD1
SH3GL1
CERCAM
UBTD2
C11orf24
FSTL1
SERPINH1
THBS2
GANAB
TXLNA
EDC3
MMP2
ADAMTS7
ADAMTS2
CLMP
MORF4L1
PDGFRB
CSPG4
FBXO22
FAM114A1
CHST14
PCOLCE
JOSD1
ITGB1
CHPF2
EXT2
CHSY1
TRIM32
PARVA
ANTXR1
ADAMTS12
FKBP9
RCN3
MVP
LOXL1
RNF4
NRAS
LGALS8
MRC2
PTRF
KIRREL
ICMT
HRAS
SEPN1
COL6A1
B4GALT2
LEPRE1
TSPAN15
FAP
COL3A1
COL16A1
KRAS
LILRB4
SPARC
COL6A2
PLOD1
COL6A3
COL5A3
LGALS9
CD276
GPX8
NRBP1
COL5A1
CD9
COL1A1
LAMA4
YKT6
COLGALT1
COL5A2
MFAP2
COL1A2
COL12A1
TNF
MXRA5
POSTN
CALU
OLFML2B
ARF4
FKBP7
PXDN
PRRX1
TR
CKAP4
LAMC1
SEC61A1
SURF4
FBN1
SNAI2
EMC1
SDF4
CTHRC1
SH3PXD2B
MMP14
ADAM12
LOXL2
TIMP2
MSANTD3
RBFOX2
BMP1
ITGB5
TPM4
CAPZB

Supplementary Table S1. The expression of CD276 RNA in different single cells.

Species	Gene	Sampled tissue	Study/sample identifier	Cluster index	Inferred cell type	Rank
Homo_sapiens	CD276	SRA705190_SRS4139633	9	Embryonic kidney	Unknown	426
Homo_sapiens	CD276	SRA709816_SRS3331832	0	ES-derived kidney organoid	Unknown	785
Homo_sapiens	CD276	SRA709816_SRS3331831	1	ES-derived kidney organoid	Unknown	908
Homo_sapiens	CD276	SRA782908_SRS3815600	16	Placenta	Unknown	928
Homo_sapiens	CD276	SRA705190_SRS4139632	1	Embryonic kidney	Unknown	932
Homo_sapiens	CD276	SRA705190_SRS4139633	4	Embryonic kidney	Unknown	949
Homo_sapiens	CD276	SRA709816_SRS3331834	2	ES-derived kidney organoid	Unknown	974
Homo_sapiens	CD276	SRA709816_SRS3331833	0	ES-derived kidney organoid	Unknown	1003
Homo_sapiens	CD276	SRA705190_SRS4139635	14	Embryonic kidney	Unknown	1046
Homo_sapiens	CD276	SRA709816_SRS3331831	8	ES-derived kidney organoid	Unknown	1090
Homo_sapiens	CD276	SRA709816_SRS3331834	0	ES-derived kidney organoid	Unknown	1146
Homo_sapiens	CD276	SRA705190_SRS4139635	7	Embryonic kidney	Unknown	1155
Homo_sapiens	CD276	SRA705190_SRS4139632	2	Embryonic kidney	Unknown	1156
Homo_sapiens	CD276	SRA704181_SRS3305831	11	Breast cancer cell line	Unknown	1198
Homo_sapiens	CD276	SRA709816_SRS3331831	11	ES-derived kidney organoid	Unknown	1252
Homo_sapiens	CD276	SRA635094_SRS2725547	8	Embryoid body	Unknown	1279
Homo_sapiens	CD276	SRA635094_SRS2725551	4	Embryoid body	Unknown	1318
Homo_sapiens	CD276	SRA709816_SRS3331832	6	ES-derived kidney organoid	Unknown	1431
Homo_sapiens	CD276	SRA704181_SRS3305832	5	Breast cancer cell line	Unknown	1485
Homo_sapiens	CD276	SRA709816_SRS3331832	7	ES-derived kidney organoid	Unknown	1499
Homo_sapiens	CD276	SRA635094_SRS2725551	9	Embryoid body	Unknown	1526
Homo_sapiens	CD276	SRA704181_SRS3305832	0	Breast cancer cell line	Unknown	1599
Homo_sapiens	CD276	SRA709816_SRS3331834	5	ES-derived kidney organoid	Unknown	1600
Homo_sapiens	CD276	SRA695134_SRS3218229	16	Alveolar rhabdomyosarcoma	Unknown	1720
Homo_sapiens	CD276	SRA795539_SRS3947512	8	Spleen	Unknown	1827
Homo_sapiens	CD276	SRA704181_SRS3305832	4	Breast cancer cell line	Unknown	1895
Homo_sapiens	CD276	SRA856537_SRS4458843	5	Unknown	Unknown	2109
Homo_sapiens	CD276	SRA695134_SRS3218229	1	Alveolar rhabdomyosarcoma	Unknown	2126
Homo_sapiens	CD276	SRA856537_SRS4458842	6	Unknown	Unknown	2127
Homo_sapiens	CD276	SRA704181_SRS3305832	10	Breast cancer cell line	Unknown	2165
Homo_sapiens	CD276	SRA795539_SRS3947512	7	Spleen	Unknown	2190
Homo_sapiens	CD276	SRA795539_SRS3947512	3	Spleen	Unknown	2194
Homo_sapiens	CD276	SRA695134_SRS3218229	6	Alveolar rhabdomyosarcoma	Unknown	2229
Homo_sapiens	CD276	SRA795539_SRS3947512	1	Spleen	Unknown	2289
Homo_sapiens	CD276	SRA704181_SRS3305830	2	Breast cancer cell line	Unknown	2341
Homo_sapiens	CD276	SRA856537_SRS4458843	2	Unknown	Unknown	2406
Homo_sapiens	CD276	SRA795539_SRS3947512	4	Spleen	Unknown	2415
Homo_sapiens	CD276	SRA856537_SRS4458843	1	Unknown	Unknown	2437
Homo_sapiens	CD276	SRA704181_SRS3305830	7	Breast cancer cell line	Unknown	2449
Homo_sapiens	CD276	SRA608611_SRS2517316	9	Lung progenitors	Unknown	2522
Homo_sapiens	CD276	SRA795539_SRS3947512	6	Spleen	Unknown	2557
Homo_sapiens	CD276	SRA795539_SRS3947512	0	Spleen	Unknown	2620
Homo_sapiens	CD276	SRA856537_SRS4458842	3	Unknown	Unknown	2651
Homo_sapiens	CD276	SRA704181_SRS3305830	0	Breast cancer cell line	Unknown	2676
Homo_sapiens	CD276	SRA856537_SRS4458844	1	Unknown	Unknown	2735

Homo_sapiens	CD276	SRA608611_SRS2517317	3	Lung progenitors	Unknown	2808
Homo_sapiens	CD276	SRA856537_SRS4458843	7	Unknown	Unknown	2904
Homo_sapiens	CD276	SRA856537_SRS4458843	6	Unknown	Unknown	2913
Homo_sapiens	CD276	SRA795539_SRS3947512	2	Spleen	Unknown	2962
Homo_sapiens	CD276	SRA856537_SRS4458843	4	Unknown	Unknown	3033
Homo_sapiens	CD276	SRA856537_SRS4458842	4	Unknown	Unknown	3065
Homo_sapiens	CD276	SRA856537_SRS4458841	2	Unknown	Unknown	3098
Homo_sapiens	CD276	SRA665712_SRS3034952	5	T cells	Unknown	3260
Homo_sapiens	CD276	SRA732346_SRS3496838	12	Endothelial cells	Unknown	3297
Homo_sapiens	CD276	SRA608611_SRS2517317	7	Lung progenitors	Unknown	3608
Homo_sapiens	CD276	SRA856537_SRS4458841	6	Unknown	Unknown	4076
Homo_sapiens	CD276	SRA709816_SRS3331834	11	ES-derived kidney organoid	Podocytes	1727
Homo_sapiens	CD276	SRA701877_SRS3279687	2	Pancreatic islets	Pancreatic stellate cells	1632
Homo_sapiens	CD276	SRA701877_SRS3279686	13	Pancreatic islets	Pancreatic stellate cells	1663
Homo_sapiens	CD276	SRA661790_SRS2995083	6	Monocyte-derived macrophages	Macrophages	676
Homo_sapiens	CD276	SRA709816_SRS3331832	17	ES-derived kidney organoid	Macrophages	924
Homo_sapiens	CD276	SRA661790_SRS2995081	2	Monocyte-derived macrophages	Macrophages	1187
Homo_sapiens	CD276	SRA661790_SRS2995081	6	Monocyte-derived macrophages	Macrophages	1375
Homo_sapiens	CD276	SRA661790_SRS2995082	7	Monocyte-derived macrophages	Macrophages	1622
Homo_sapiens	CD276	SRA661790_SRS2995084	5	Monocyte-derived macrophages	Langerhans cells	1403
Homo_sapiens	CD276	SRA608611_SRS2517316	1	Lung progenitors	Hepatocytes	3327
Homo_sapiens	CD276	SRA703206_SRS3296614	5	Colon (Ulcerative Colitis)	Fibroblasts	1392
Homo_sapiens	CD276	SRA782908_SRS3815600	15	Placenta	Fibroblasts	1574
Homo_sapiens	CD276	SRA782908_SRS3815600	17	Placenta	Fibroblasts	1604
Homo_sapiens	CD276	SRA676027_SRS3102136	1	Embryonic stem cell line	Fibroblasts	1786
Homo_sapiens	CD276	SRA676027_SRS3102144	1	Embryonic stem cell line	Fibroblasts	1796
Homo_sapiens	CD276	SRA703206_SRS3296614	14	Colon (Ulcerative Colitis)	Fibroblasts	1846
Homo_sapiens	CD276	SRA700673_SRS3270097	5	Brain	Fibroblasts	1993
Homo_sapiens	CD276	SRA700673_SRS3270097	12	Brain	Fibroblasts	2309
Homo_sapiens	CD276	SRA782908_SRS3815606	2	Decidua	Fibroblasts	2636
Homo_sapiens	CD276	SRA700673_SRS3270097	3	Brain	Fibroblasts	2657
Homo_sapiens	CD276	SRA782908_SRS3815606	0	Decidua	Fibroblasts	2932
Homo_sapiens	CD276	SRA709816_SRS3331831	3	ES-derived kidney organoid	Endothelial cells	539
Homo_sapiens	CD276	SRA709816_SRS3331832	5	ES-derived kidney organoid	Endothelial cells	612
Homo_sapiens	CD276	SRA709816_SRS3331833	6	ES-derived kidney organoid	Endothelial cells	652
Homo_sapiens	CD276	SRA646572_SRS2833947	24	Human embryo forebrain	Endothelial cells	3827
Homo_sapiens	CD276	SRA665712_SRS3034953	34	T cells	Basal cells	902
Homo_sapiens	CD276	SRA665712_SRS3034951	9	T cells	Basal cells	1042
Homo_sapiens	CD276	SRA665712_SRS3034953	8	T cells	Basal cells	1344
Homo_sapiens	CD276	SRA676027_SRS3102144	9	Embryonic stem cell line	Basal cells	1637
Homo_sapiens	CD276	SRA729910_SRS3474727	11	Lung airway epithelial cells	Basal cells	2262
Homo_sapiens	CD276	SRA760933_SRS3693061	1	Hepatocyte-derived liver	Basal cells	2460

				progenitor-like cells		
Homo_sapiens	CD276	SRA691388_SRS3173556	12	Breast epithelium	Basal cells	2609
Homo_sapiens	CD276	SRA729910_SRS3474726	8	Lung airway epithelial cells	Basal cells	2937
Homo_sapiens	CD276	SRA709816_SRS3331834	12	ES-derived kidney organoid	Basal cells	2993
Homo_sapiens	CD276	SRA729910_SRS3474726	4	Lung airway epithelial cells	Basal cells	3192
Homo_sapiens	CD276	SRA608611_SRS2517316	3	Lung progenitors	Basal cells	3440
Homo_sapiens	CD276	SRA729910_SRS3474727	7	Lung airway epithelial cells	Basal cells	3566
Homo_sapiens	CD276	SRA729910_SRS3474726	1	Lung airway epithelial cells	Basal cells	3666
Homo_sapiens	CD276	SRA729910_SRS3474726	9	Lung airway epithelial cells	Basal cells	3679
Homo_sapiens	CD276	SRA729910_SRS3474726	2	Lung airway epithelial cells	Basal cells	3753
Homo_sapiens	CD276	SRA729910_SRS3474727	1	Lung airway epithelial cells	Basal cells	4009
Homo_sapiens	CD276	SRA640903_SRS2780388	5	Melanoma	Basal cells	4298
Homo_sapiens	CD276	SRA729910_SRS3474726	6	Lung airway epithelial cells	Basal cells	4464
Homo_sapiens	CD276	SRA729910_SRS3474727	0	Lung airway epithelial cells	Basal cells	4599
Homo_sapiens	CD276	SRA728025_SRS3454421	11	Inflamed area of colon	Basal cells	4644
Homo_sapiens	CD276	SRA728025_SRS3454422	9	Colon	Basal cells	4758
Homo_sapiens	CD276	SRA728025_SRS3454423	9	Colon	Basal cells	4831

The online database PanglaoDB(16) was used to explore the expression of CD276 RNA in different single cells.

Supplementary Table S2. Cancer Types and Abbreviations Table

**Abbreviation**	**Full name of cancers**
BLCA	Bladder urothelial carcinoma
BRCA	Breast invasive carcinoma
CHOL	Cholangiocarcinoma
COAD	colon adenocarcinoma
ESCA	Esophageal carcinoma
GBM	Glioblastoma multiforme
HNSC	Head and neck squamous cell carcinoma
KICH	Kidney chromophobe
KIRC	Kidney renal clear cell carcinoma
KIRP	Kidney renal papillary cell carcinoma
LIHC	Liver hepatocellular carcinoma
LUAD	Lung adenocarcinoma
LUSC	Lung squamous cell carcinoma
PRAD	Prostate adenocarcinoma
READ	Rectum adenocarcinoma
STAD	Stomach adenocarcinoma
THCA	Thyroid carcinoma
UCEC	Uterine Corpus Endometrial Carcinoma
DLBC	Lymphoid neoplasm diffuse large B-cell lymphoma
LGG	Brain lower grade glioma
PAAD	Pancreatic adenocarcinoma
TGCT	Esticular germ cell tumors
THYM	Thymoma
UCS	Uterine carcinosarcoma
ACC	Adrenocortical carcinoma
CESC	Cervical squamous cell carcinoma and endocervical adenocarcinoma
LAML	Acute myeloid leukemia

OV	Ovarian serous cystadenocarcinoma
PCPG	Pheochromocytoma and paraganglioma
